# Remineralizing Potential of Natural Nano-Hydroxyapatite Obtained from *Epinephelus chlorostigma* in Artificially Induced Early Enamel Lesion: An In Vitro Study

**DOI:** 10.3390/nano12223993

**Published:** 2022-11-12

**Authors:** Ashwathi Mathirat, Pandurang Appana Dalavi, Ashwini Prabhu, Yashaswini Devi G.V., Sukumaran Anil, Kalimuthu Senthilkumar, Gi Hun Seong, Sharan S. Sargod, Sham S. Bhat, Jayachandran Venkatesan

**Affiliations:** 1 Department of Pediatric and Preventive Dentistry, Yenepoya Dental College, Yenepoya University, Mangalore 575018, Karnataka, India; 2 Biomaterials Research Laboratory, Yenepoya Research Centre, Yenepoya (Deemed to be University), Deralakatte, Mangalore 575018, Karnataka, India; 3 Department of Dentistry, Oral Health Institute, Hamad Medical Corporation, Doha 3050, Qatar; 4 College of Dental Medicine, Qatar University, Doha 2713, Qatar; 5 Central Research Laboratory, Swamy Vivekananda Medical College Hospital and Research Institute, Namakkal 637205, Tamilnadu, India; 6 Department of Bionano Engineering, Center for Bionano Intelligence Education and Research, Hanyang University, Ansan 426-791, Korea

**Keywords:** fishbone, demineralization, nano hydroxyapatite, remineralization

## Abstract

Dental caries is a common problem in adolescents, leading to permanent loss of teeth or cavitation. Caries is a continuous process wherein demineralization and remineralization occur regularly. Hydroxyapatite (HA) is one of the most biocompatible and bioactive materials, as it closely resembles the mineral composition of teeth. The present study deals with isolating hydroxyapatite from fish bone (*Epinephelus chlorostigma*) by alkaline hydrolysis and thermal calcination. The isolated nano HA was characterized using FT-IR, XRD, TGA, FE-SEM-EDX, and HR-TEM analysis. The nano HA isolated by alkaline hydrolysis is nontoxic, and the cells are viable. The isolated HA enhances the proliferation of L929 cells. The remineralization potential of the extracted nano HA was evaluated in healthy premolars by DIAGNOdent/laser fluorescence quantification, surface microhardness test, and SEM-EDX analysis. Surface morphological observations in SEM and EDX analyses show that thermally calcined HA and alkali-treated HA can induce mineralization and deposit minerals. Therefore, HA obtained from *Epinephelus chlorostigma* could be a potential biomaterial for treating early caries.

## 1. Introduction

Dental caries is considered the most significant global oral health burden and is a dynamic process with multiple remineralization steps. The reversal of incipient or early enamel caries plays an essential role in prevention and leads to the repair of lesions. Biocomposites containing hydroxyapatite, casein phosphopeptides, amorphous calcium phosphate, and other antibacterial agents are mainly used to stop caries and regenerate teeth [1,2,3,4]. Nano hydroxyapatite (HA) is a thermodynamically stable ceramic with the chemical formula Ca_10_(PO_4_)_6_(OH)_2_. HA is used in various applications, including orthopedics, dentistry, drug delivery, the food industry, catalysis, fertilizer, and heavy metal removal [5,6]. HA is an extensively studied bone graft substitute material and attracts much attention in dentistry due to its excellent biocompatibility, non-toxicity, mechanical strength, and non-immunogenicity. HA has been used in dentistry for remineralization and implant and cell adhesion enhancement. In addition, coating metal implants with HA is explicitly used because HA promotes osseointegration [7,8]. Various methods are known for the synthesis of HA, including the precipitation method, gel diffusion method, sol-gel method, hydrothermal method, electrodeposition method, pyrolysis, solid-state synthesis, mechanochemical method, hydrolysis method, emulsion method, sonochemical method, and so on [9,10,11,12,13]. However, these synthetic chemical methods may require hazardous chemicals and reagents that lead to environmental threats. In addition, the synthetic form requires drastic physical and chemical processes and is expensive. Hence, researchers focus on eco-friendly and cost-effective methods to develop HA using the green method [14,15].

Various environmentally friendly approaches have been used to produce HA. Currently, researchers focus on natural sources, including plants, animals, biogenic materials, and aquatic sources, to develop HA. HA powder derived from natural sources such as fish bones is inexpensive and abundant. The most critical parameters responsible for forming HA nanoparticles are the temperature and duration of heat treatment and other synthetic changes, such as alkali treatment or treatment with subcritical water [16,17]. Our previous study isolated HA from fish bones using the alkaline hydrolysis method. Physicochemical characterization confirms the formation of nano HA and the particle size of nano HA ranges [18]. Thermal calcination and alkaline hydrolysis have been used to isolate natural HA from animal bones. The traditional method of producing HA is thermal calcination. The alkaline hydrolysis method is easy to use and has several advantages, including making nanostructured particles, carbonated HA powder, and an environmentally friendly process [18]. Hamour fishbone is commonly described for several fishes, including the brown-spotted reef cod (*Epinephelus chlorostigma*). The name has been given to several closely related fish species in the Persian Gulf and on the southwest coast of India. It is a relatively widespread species inhabiting reefs in shallow and deep waters.

In the present study, HA was prepared from fish bones of *E. chlorostigma* by alkaline hydrolysis and thermal calcination, and the prepared material was characterized using various characterization techniques. Furthermore, the biocompatibility of nano HA with L929 cells was tested for further biological and biomedical applications. Furthermore, an artificial caries lesion evaluated the remineralizing potential of isolated HA. The current study aims to understand the remineralization potential of fish bone-derived HA in artificially induced early enamel lesions. On early lesions of enamel, HA displays remineralizing effects, and it will be an excellent substance with widespread dental applications that can assist in repairing and preventing tooth enamel erosion.

## 2. Materials and Methods

The fish bones from *Epinephelus chlorostigma* were brought from Mangalore. Sodium hydroxide was purchased from Merck in Mumbai, India. DMEM (Dulbecco’s Modified Eagle Medium), PBS (Phosphate Buffered Saline) and L-glutamine were procured from Gibco, Thermo Scientific, Waltham, MA, Boston USA. The L929 cell line was obtained from The National Centre for Cell Science (NCCS) in Pune. The study included 51 healthy human premolars extracted for orthodontic reasons. In addition, the study consists of healthy quasi-teeth without restorations, while teeth with deformities, abnormalities, or fractures were excluded. The in vitro experiments were performed according to the Yenepoya (Deemed to be University) ethical committer 2 permission number (YEC2/206).

### 2.1. Preparation of the Fish Bone Powder

The fish bones were washed in hot water for two days to remove any remnants of flesh or skin. Next, the cleaned bones were mixed with 1.0% sodium hydroxide and acetone (the ratio of bones and sodium hydroxide (solid/liquid) was kept at 1:50) to remove proteins, oils, fats, and other chemical compounds. The bones obtained were washed well before being pulverized in a mortar and pestle and dried at 60 °C for 24 h.

### 2.2. Alkaline Hydrolysis Method

Then, 2 g of groundfish bone was treated with 2 M sodium hydroxide at 250 °C for 5 h. This procedure was carried out several times to ensure that the organic components were adequately removed. The mixture was then filtered in a suction pump with constant washing with water until it was neutral. Finally, the product obtained was dried in an oven at 100 °C.

### 2.3. Thermal Calcinations Method

First, 2 g of fish bone was placed in a silicate crucible in an electric muffle furnace and heated to 600 °C for 4 h. After calcination, the silicate crucible was allowed to cool at room temperature, and the material obtained was used as HA.

### 2.4. Chemical Characterization of Nano-Hydroxyapatite from Fishbone

The isolated HA stretching frequencies were investigated using a Shimadzu Fourier transform infrared spectrophotometer (FT-IR) with a single reflection ATR accessory (Shimadzu, Kyoto, Japan) in a spectral range of 4000–650 cm^−1^. Thermogravimetric analysis (TGA) was performed (STA 600 Perkin Elmer, Waltham, MA, USA). The morphology of the isolated HA was examined using field emission scanning electron microscopy (FE-SEM) (Carl Zeiss, Oberkochen, Germany). Energy-dispersive X-ray spectroscopy (EDS; Oxford instrumentation, ZEISS, Begbroke Science Park, UK) was used to confirm the presence of the elements calcium (Ca) and phosphorus (P) in the material. Powder X-ray diffraction (P-XRD) patterns confirmed the isolated HA by matching the crystallographic phases. P-XRD was documented with a Bruker D8 (Ettlingen, Germany) diffractometer using the Cu K = 1.5406 radiation range of 20°–80° with a step size of 0.01. Transmission electron microscopy analyses were performed (JEOL, JEM 2100, Dearborn Road Peabody, MA, USA). SAED analysis was also performed to evaluate the crystallinity of the isolated nano HA (JEOL, 6390LA/OXFORD XMX N, Dearborn Road Peabody, MA, USA).

### 2.5. Biocompatibility Assay of Isolated HA

MTT assay was performed to investigate the cell proliferation and cytotoxicity of the isolated HA. L929 cells were cultured in Dulbecco’s modified Eagle’s medium containing 10% FBS and 1% antibiotic-antimycotic solution. The cells were maintained at 37 °C and 5% CO_2_ in a humidified environment. Cells were then seeded at a density of 5000 cells/well on 96-well microtiter plates and allowed to adhere to alkaline HA. Next, alkaline HA at different concentrations, i.e., 10 µg/mL, 50 µg/mL, 100 µg/mL, 250 µg/mL and 500 µg/mL, were treated, and the cells were incubated for 24 h at 37 °C and 5% CO_2_ in a humidified atmosphere. The medium was then removed, and an MTT reagent (1 mg/mL) was added and incubated for 4 h at 37 °C and 5% CO_2_ in a humidified atmosphere. Finally, DMSO was added, absorbance at 570 nm was recorded with a multimode microplate reader (FluoSTAR Omega, BMG Labtech, Offenburg, Germany), and cytocompatibility was calculated.

### 2.6. Hoechst 33342 Staining Assay

Cell growth was visualized using the Hoechst 33342 staining assay. First, L929 cells were seeded at a density of 5000 cells/well on 96-well plates and incubated for 24 h at 37 °C and 5% CO_2_. Then, alkali-treated nano HA was added at concentrations of 10 μg/mL and 500 μg/mL and incubated for 24 h at 37 °C and 5% CO_2_ in a humidified atmosphere. The medium was then removed, and the cells were stained with a fluorescent binding dye, i.e., a Hoechst staining solution (5 μg/mL), for 10 min under dark light conditions. Subsequently, the excess staining solution was removed, and the cells were washed three times with PBS. The cells were then coated with PBS, and images were taken in the blue channel at 20× magnification using a fluorescence imager (ZOE, BioRad, CA, USA).

### 2.7. Specimen Preparation

The study included 51 healthy human premolars taken from the Department of Oral and Maxillofacial Surgery for orthodontic procedures. Teeth with abnormalities, defects, or fractures were excluded from the study. The teeth were thoroughly cleaned and stored in saline (0.9% sodium chloride solution) until needed. The crown was removed from the root and cut into two pieces before being placed on Plexiglas with a high-speed diamond disc. Finally, the ground surface was polished with a pumice polishing paste. The teeth were painted with an acid-resistant nail varnish that exposed a 2 mm × 2 mm window in the Centre of the buccal and lingual surfaces of the tooth areas. Before the nail varnish was applied, each specimen was dried with compressed air. The baseline values of the section within the window were assessed with DIAGNOdent/Laser fluorescence (LF) and the microhardness of the surface for all samples.

### 2.8. Demineralization of Tooth

Each sample was immersed for 48 h in 15 mL of a demineralizing solution containing 2.2 mM CaCl_2_, 0.05 mM lactic acid, and 0.5 ppm fluorine. The pH of the solution was adjusted to 4.5 with 50% NaOH. Ashy, white, caries-like lesions had formed on the surfaces of the samples. Subsequently, demineralization was measured with the laser fluorescence technique (DIAGNodent), the surface microhardness test, and SEM with the EDX analysis test. Diagnodent values and surface microhardness values were determined as previously described.

### 2.9. Preparation of Treatment Slurries

Alkaline HA and thermal HA were measured and deposited in separate beakers. Each substance was incorporated by hand into the water, then stirred with a stir bar and stir plate (rpm = 400) to achieve the uniform dispersion of the active ingredient until the slurry was uniform in color and dense in texture.

### 2.10. Remineralization of Tooth

The control (GC Tooth Mousse) and the treatment groups, thermal HA and alkaline HA, were used for remineralization. To remineralize carious lesions in enamel, topical application in the form of a slurry) of thermal HA, alkaline HA and GC Tooth Mousse was performed and allowed to dry. All of the samples were soaked in artificial saliva for 10 days after receiving the respective group treatment. The artificial saliva was prepared with a pH of 7 using the reported methodology [19].

### 2.11. Laser Fluorescence (DIAGNOdent) Method

Laser fluorescence (DIAGNOdent), a non-invasive assessment method based on the red end of the electromagnetic spectrum with a light wavelength of 655 nm, was used (DIAGNOdent or Diagnodent pen, KaVo, Biberach an der Riss, Germany). Probe tip B was selected. Before the examination, the laser device was calibrated against a porcelain reference object and recalibrated after measuring all samples’ peak values within the window range.

### 2.12. Surface Microhardness Evaluation Using the Vickers Micro Hardness Tests (VMHTs)

The Vickers hardness number has been used to determine the micro-hardness of the surface. In most cases, Vickers microhardness tests (VMHTs) are used to determine the hardness of materials over various microhardness test loads (typically 1–1000 g). Under the microscope, the square shape of the residual indentation produced by VMHTs is easy to measure. The Vickers hardness number was used to determine the surface microhardness of the samples before demineralization. Each model is impressed with a single force and holding time at each study stage. The average results of the measurements were used to determine the surface microhardness (SMH).

### 2.13. Surface Analysis of Remineralized Teeth after Treatment of HA

The remineralized samples were examined using SEM at 0.5–30 kV. SEM was used to analyze the samples’ surface texture patterns and melt density. EDX analysis was also used to determine the calcium (Ca) and phosphate (P) concentrations in the surface melt. Finally, the ratio of calcium (Ca) to phosphate (P) was calculated to confirm the samples’ remineralization.

### 2.14. Statistical Analysis

Graphpad Prism 8 and Origin Lab software were used to draw the figures and for further interpretation. Three independent experiments were performed for the cell-related assays, and the standard deviations were plotted to obtain significant values. The data were analyzed using SPSS version 22.0 (SPSS Inc., Chicago, IL, USA).

## 3. Results and Discussion

**Figure 1:**
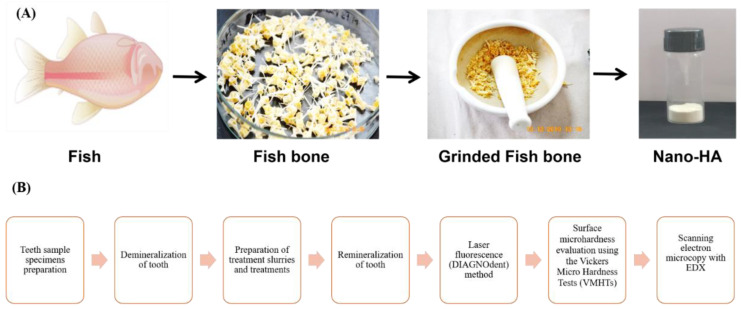
(**A**) Schematic diagram of the preparation of nano HA powder from the fishbone. The two different methodologies, thermal calcination and alkaline hydrolysis, were utilized to isolate HA from the fishbone, and (**B**) Summary of processes involved in demonstrating the remineralization potential of the isolated nano HA.

Nanotechnology is emerging as one of the most revolutionary approaches to developing nanoscale materials [20]. In recent years, researchers have focused on nanotechnology because of the dramatic change in material properties at the nanoscale due to the larger surface-to-volume ratio [21]. In terms of shape and crystal structure, the nanoparticles are identical to the apatite crystals in tooth enamel. Some report has shown that HA has excellent potential for repairing and regenerating tooth enamel [22]. The research results show that HA can be used in clinical oral surgery. These include the treatment of periodontal bone abnormalities, the filling of bone deficits after cyst removal and root tip resections, and the augmentation of atrophic alveolar ridges during the removal of dental implants [23]. In addition, tooth decay can also be prevented by adding HA to toothpaste, which binds with proteins, plaque, and bits of bacteria. They act as a cushion and fix tiny imperfections on the enamel surface. Due to their remarkable biocompatibility and bioactivity, HA can also improve the properties of materials used for dental restorations [24,25,26]. Hence, we isolated nano HA from the fish bone using thermal calcination and alkaline hydrolysis process (Figure 1A) and followed the remineralization process with the isolated HA in vitro. FT-IR and XRD analysis were used to determine the functional group and purity of the isolated HA. The XRD was used to compare the JCPDS data with the isolated HA to understand the phase and purity of the product. TGA analysis was performed to understand the complete removal of the organic portions from the fishbone.

### 3.1. Fourier Transform-Infrared (FT-IR) Spectroscopy

**Figure 2:**
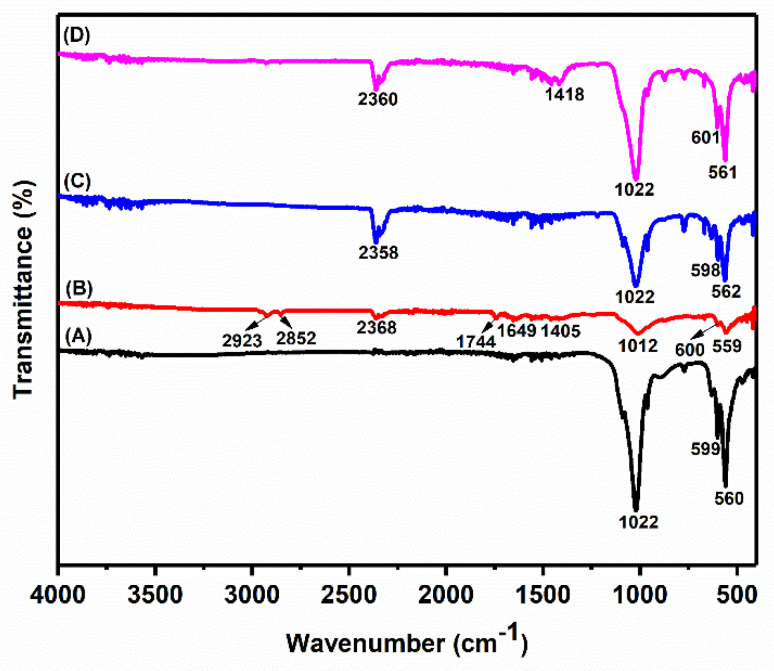
FT-IR spectrum of (A) Synthetic HA, (B) Raw fish bone, (C) Thermal HA, and (D) Alkali treated nano HA.

FT-IR analysis was carried out to determine the functional groups in the materials in the 400–4000 cm^−1^ using a single reflection ATR method. Figure 2 shows the spectra of (A) synthetic HA, (B) raw fish bone, (C) thermal HA, and (D) alkaline HA. In spectrum (A), a characteristic peak at 599 and 560 cm^−1^ belongs to the asymmetric bending mode of the P-O group [27]. On the other hand, a sharp peak at 1022 cm^−1^ belongs to the symmetrical stretching of the phosphate group (PO_4_^3−^). Furthermore, in spectrum (B), the characteristic peaks at 1649 and 1744 cm^−1^ are associated with the collagen moieties present in the material. In addition, the C-H stretching groups show a pronounced peak at 2852 and 2923 cm^−1^. Furthermore, peaks at 1405 cm^−1^ arise from the out-of-plane bending mode of the carbonate group present in the raw fishbone.

All of the peaks at 560 cm^−1^, 599, and 1022 cm^−1^ belong to PO_4_^3−^ groups that are individually consistent from synthetically obtained HA with isolated HA from *E. chlorostigma* fish bone. A characteristic peak at 1418 cm^−1^ in the spectra (D) belongs to the carbonate group (–O_3_^−2^), which means that we have carbonated HA by alkaline hydrolysis [28,29,30].

### 3.2. X-ray Diffraction (XRD) Analysis

XRD analyses were performed to confirm the purity of the isolated HA and the HA isolated from *E. chlorostigma* bone by comparing the crystallographic planes with the Joint Committee on Powder Diffraction Standards for hydroxyapatite (JCPDS) standard data from HA. All of the data were collected in the range of 2θ values from 10° to 80°. From the JCPDS standard data of HA, one crystallographic plane (0 0 2), (2 1 1), (1 1 2), (3 0 0), (2 0 2), (3 1 0), (2 2 2), (2 1 3), (3 2 1), and (0 0 4) belongs to the pure HA [31].

**Figure 3:**
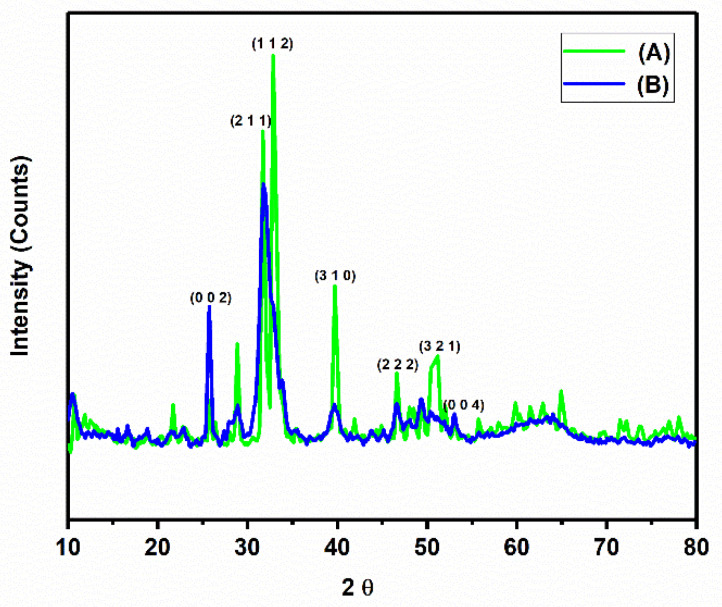
P-XRD patterns of the isolated nano HA, graph (A) thermally calcined nano HA, and graph (B) alkali-treated nano HA.

Figure 3 (A) for the thermally calcined nano HA and spectrum (B) for the alkali-treated nano HA. In spectrum (A) and spectrum (B), the crystallographic planes (0 0 2), (2 1 1), (3 1 0), (2 2), (3 2 1), and (0 0 4) were observed, and the diffraction indices are consistent with the JCPDS data [29,30]. Sharp and intense peaks were obtained in the hydrothermally calcined nano HA, indicating the formation of higher crystallinity [32]. The data obtained in our study are comparable to the findings of various research articles. Mustafa et al. isolated HA from fish bone waste using the calcination method. The intensity of the peaks increased with increasing calcination temperature, indicating the formation of pure HA [33]. Pal et al. have extracted HA from Lates calcarifer fish using different calcination temperatures. XRD analysis shows that at 400 °C, almost all organic components were removed. The calcination of the raw material at above 800 °C resulted in the formation of tricalcium phosphate with pure HA [34]. Pallela et al. have used polymer-assisted isolation of HA from fish bones. The addition of polymers to fish bones has a significant effect on the isolation of HA [35].

### 3.3. Thermogravimetric (TGA) Analysis

**Figure 4:**
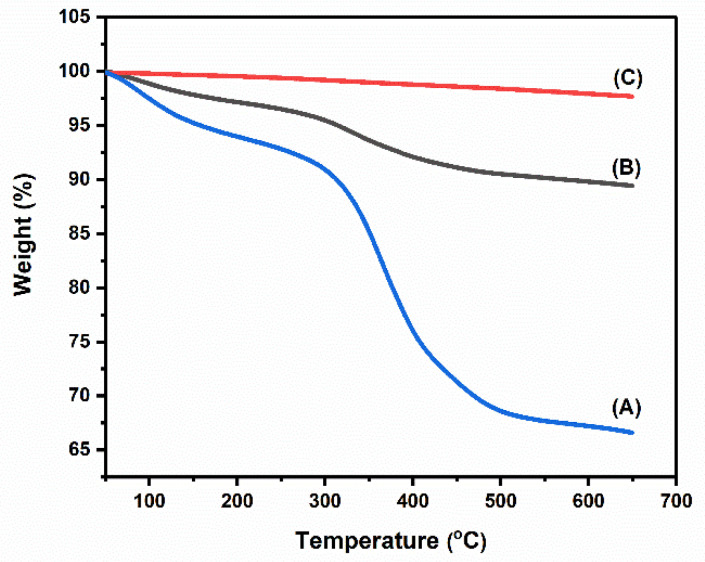
Thermogravimetric analysis of (A) raw fish bone, (B) alkali-treated nano HA, and (C) thermally calcined nano HA.

Thermogravimetric analysis was carried out to determine the isolated material’s thermal stability. The thermogravimetric analysis of the isolated HA in the range of 50–700 °C is shown in Figure 4, spectrum (A) Raw fish bone, (B) Alkali treated nano HA, and (C) thermal HA. In spectrum (B), there is a slight depletion at 360 °C, possibly due to a trace of collagen moieties. In diagram 4 (A), there is a bend in a peak at almost 360 °C due to the presence of collagen and organic components. Figure 4C shows no depletion point at any temperature. The TGA analysis thus confirms that the collagen components and other impurities were eliminated by the thermal calcination and the alkaline treatment [36,37].

### 3.4. Field-Emission Scanning Electron Microscopy (FE-SEM) with Energy-Dispersive X-ray Spectroscopy (EDX) Analysis

**Figure 5:**
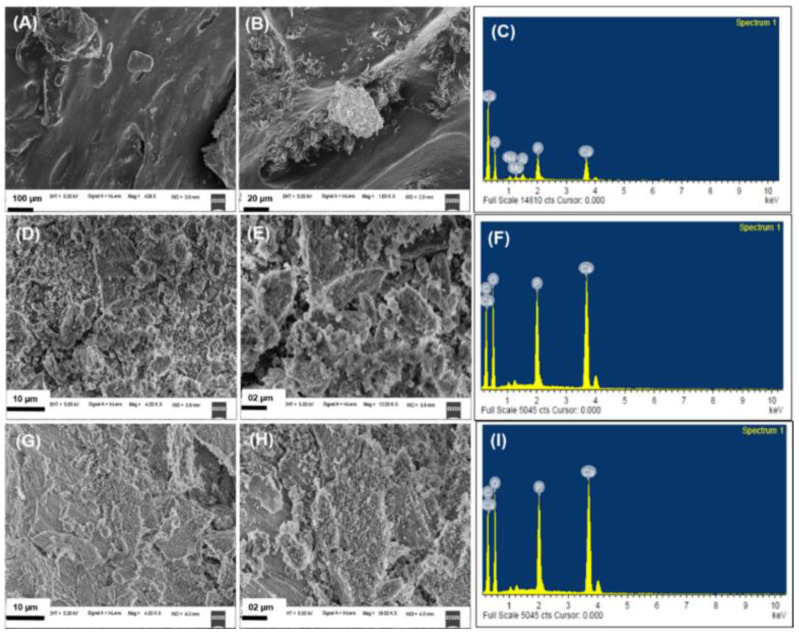
FE-SEM-EDX images (**A**,**B**) of raw fishbone, images (**D**,**E**) for alkali-treated nano HA, images (**G**,**H**) for thermally calcined nano HA, and images (**C**,**F**,**I**) EDX images of raw fishbone, alkali-treated nano HA and thermally calcined nano HA, respectively.

Figure 5A,B,D,E,G,H depict the FE-SEM images of raw bone, alkali-treated nano HA, and thermal calcination-derived nano HA, respectively. Figure 5A,B shows the smooth morphology with the presence of collagen moieties and embedded HA nanocrystals. However, nano HA derived from alkaline hydrolysis and thermal calcination methods produced nano HA by removing organic moieties. Hence, the changes in the morphology were observed (Figure 5D,E,G,H) when compared to the raw fish bone. Next, Figure 5C,F,I are the EDX images of raw fish bone, alkali-treated nano HA, and thermally calcined nano HA, respectively. The presence of Ca and P was confirmed using EDX analysis. The Ca/P ratio of pure HA is 1.67. Further, the EDX analysis confirms that alkaline-treated nano HA has a 1.61 Ca/P ratio, and the calcination methodology developed nano HA has a 1.46 Ca/P ratio [31,38]. Shi et al. reported that the Ca/P ratio of isolated HA was 1.47, 1.88, and 1.51 for rainbow trout, cod, and salmon fish species, respectively [39].

Panda et al. reported that the isolated HA has a 1.6 Ca/P ratio [40]. In another study, Zeng et al. isolated HA from various fish bone species, including rainbow trout, cod, and salmon fish. The thermal calcination method was used to isolate nano HA. From the EDX analysis, the isolated nano HA has Ca/P ratios of 1.47, 1.88, and 1.51 for the rainbow trout, cod, and salmon fish species, respectively [41]. Our results of the Ca/P ratio of isolated nano HA using thermal calcination and alkaline hydrolysis method agree with the literature. It can be observed that the Ca/P ratio changes from one species to another. Furthermore, the thermal calcination method changes the Ca/P ratio significantly compared to the alkaline hydrolysis method. The thermal calcination method produces lower Ca/P values.

### 3.5. High-Resolution Transmission Electron Microscopy (HR-TEM) Analysis

**Figure 6:**
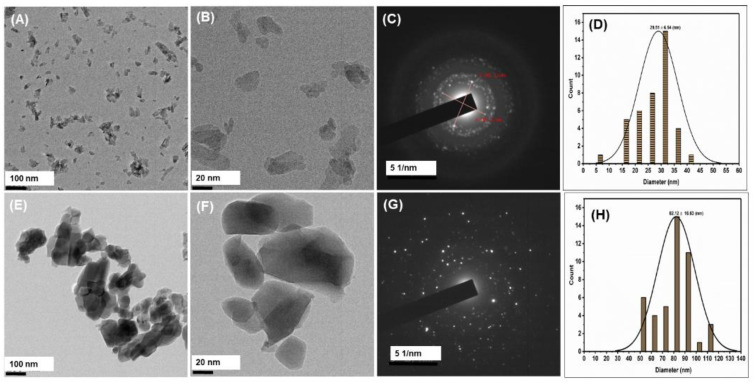
HR-TEM images (**A**,**B**) for alkali-treated nano HA, respectively, and images (**E**,**F**) for thermally calcined nano HA, respectively, and images (**C**,**G**) SAED pattern of alkali-treated nano HA and thermally calcined nano HA, respectively, and images (**D**,**H**) are histogram obtained from the particle size distribution of the alkali-treated nano HA and thermally calcined nano HA, respectively.

Figure 6A,B shows the HR-TEM images of alkali-treated nano HA at 100 and 20 nm magnification, respectively. Figure 6C shows the SAED pattern of alkali-treated nano HA. Furthermore, Figure 6E,F shows the HR-TEM images of thermally calcined nano HA at 100 and 20 nm magnification, respectively. Figure 6G shows the SAED pattern of thermally calcined nano HA. Figure 6D,H are the histograms of alkali-treated nano HA and thermally calcined nano HA. The results show that the nano HA isolated by the alkaline hydrolysis method has an average particle size of 29.5 nm. In comparison, the nano HA isolated by the thermally calcined method has an average particle size of about 82.12 nm. Furthermore, the bright white spots in the SAED analysis images indicate that the particles are crystalline [18,42].

### 3.6. Biocompatibility Study with Isolated HA

**Figure 7:**
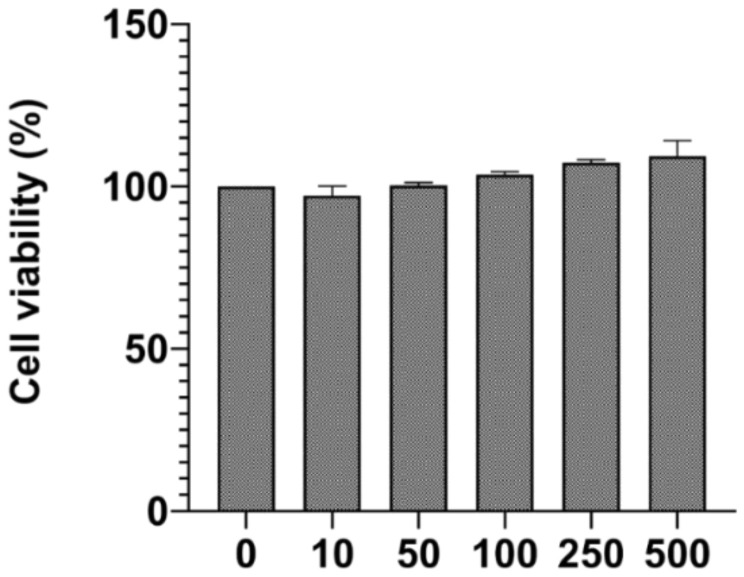
MTT assay results with L929 cells at different concentrations of alkali-treated nano HA, standard deviation measured using three independent values.

Figure 7 illustrates the percentage cell viability of L929 fibroblast cells. From the MTT assay results, all concentrations of alkali HA, i.e., 10 µg/mL, 50 µg/mL, 100 µg/mL, 250 µg/mL, and 500 µg/mL, are biocompatible. The MTT method has been extensively used to measure the materials’ biocompatibility or toxicity [43]. The isolated HA can play a promising role in the biomedical field. Pal et al. have performed cell interaction studies with human osteoblast-like MG-63 cells, confirming that isolated HA was biocompatible with MG-63 cells. The data obtained suggest that the isolated HA from the fish Lates calcarifer has promising applications for bone tissue engineering [34]. In vitro cell interaction studies with MC3T3-E1 osteoblast cells show that the isolated HA was biocompatible. The HA isolated from the fishbone of *Thunnus obesus* can be used in orthopedic clinical treatment [44]. Furthermore, in vitro cell interaction studies with mesenchymal stem cells show that the extracted nano HA has promising applications in tissue engineering [40].

Afzal et al. investigated the biocompatibility of the HA-alumina-zirconia biocomposition with L929 fibroblast cells and Saos-2 osteoblast cells. The results of the MTT test show that the biocomposition produced was biocompatible and can promote cell proliferation. The HA-alumina-zirconia biocomposite can be used as a bone implant [45]. Dubey et al. have studied cellular response studies of the L929 mouse fibroblast cells and Saos-2 osteoblast cells with the developed HA with perovskite (CaTiO_3_) biocomposites. MTT results show that cells are well adhered, and cell density increases with incubation time. Findings imply that HA-CaTiO_3_ biocomposite is nontoxic and cytocompatible with L929 mouse fibroblast cells and Saos-2 osteoblast cells [46]. Our study’s MTT assay data showed that alkali-treated nano HA was biocompatible with the L929 cells.

### 3.7. Hoechst 33342 Staining Assay

**Figure 8:**
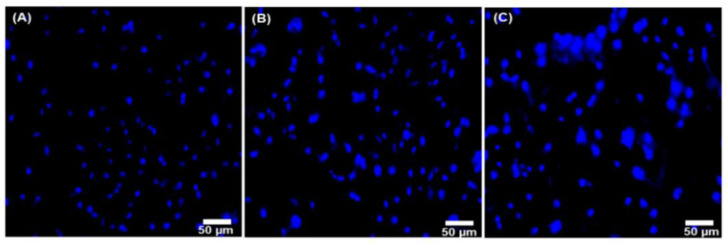
Hoechst 33342 staining images of alkaline treated nano HA at different concentrations, image (**A**) control (**B**) 10 µg/mL, and (**C**) 500 µg/mL, Scale bar—50 μm.

The Hoechst 33342 staining assay studied the growth and proliferation of the L929 cells. Figure 8A–C shows the Hoechst 33342 staining images of control, alkaline-derived nano HA treated with 10 µg/mL, and 500 µg/mL, respectively. Cell growth and proliferation were observed at both concentrations, indicating that isolated alkali nano-HA can enhance the proliferation of the L929 cells [44,47].

### 3.8. Remineralization Efficiency of the Isolated HA

**Figure 9:**
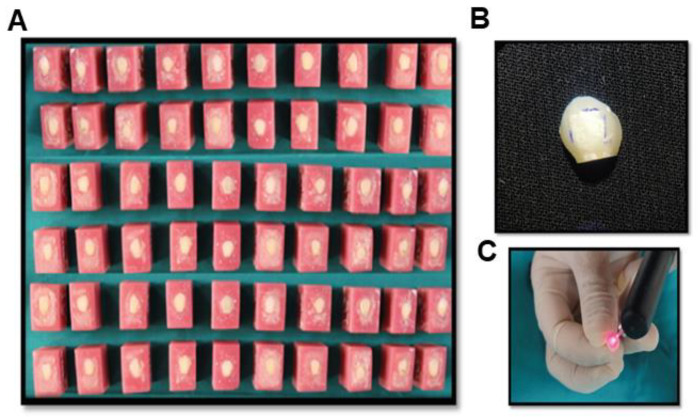
(**A**) Natural teeth mounted acrylic block, the prepared teeth for the experiment, (**B**) demineralized tooth specimen, and (**C**) assessment using DIAGNOdent instrument.

**Figure 10:**
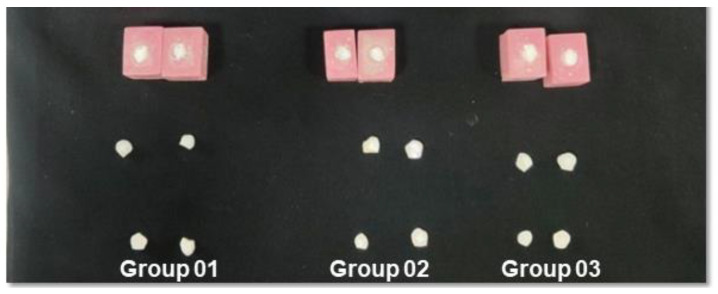
Three different specimen groups were utilized in the present study to assess the remineralization potential of the developed materials. Group 1 contains the tooth GC-Tooth-Mousse commercially available product, and Group 2 comprises thermal HA from the fish bone; Group 3 involves the alkaline HA from the natural fish bone.

The remineralization ability of the material gives an idea of whether the material can restore and regenerate osseous tissue or not. Hence, to assess the effectiveness of the isolated nano HA powder on enamel remineralization, we performed DIAGNOdent/Laser fluorescence quantification and surface microhardness tests on the enamel specimens (Figure 9 and Figure 10). Furthermore, in the study, enamels were incubated in artificial saliva. Finally, SEM/EDX analysis was used to confirm the remineralization process [48].

### 3.9. Remineralizing Potential Assessed Using DAIAGNOdent (Laser Fluorescence-LF)

The early stages of dental caries were examined by measuring the laser fluorescence (LF) readings of the enamel surface. LF readings provide precise information about the formation of dental caries. LF readings can distinguish between a healthy tooth and a tooth with a carious lesion by its fluorescing capacity. Changes in the properties of mineral components in demineralized teeth, such as reflection, transmission, and color absorption, change the LF reading, causing the value to vary from healthy teeth, aiding in caries detection [49,50]. Before demineralization, the tested groups showed low LF values, i.e., nearly 2; however, after demineralization, higher LF values were observed due to the loss of mineral content. After remineralization, a reduction in the LF values was observed due to the inducement of the mineralization. Thermal HA shows an LF value of about 4.52 ± 1.17, and alkaline offers an LF value of about 6.17 ± 0.088. This data reveals that alkali-treated nano HA and thermally calcined nano HA can promote mineral deposition.

### 3.10. Remineralizing Potential Assessed Using Surface Microhardness

Microhardness testing is commonly used in dentistry because it provides insight into surface changes in the enamel that contribute to tooth deterioration. Because of its great precision and quantitative measurement capabilities, the Vickers hardness technique is most often employed to calculate the surface hardness of enamel.

The Vickers hardness technique is utilized to analyze the demineralization and remineralization of the enamel [51,52,53]. All groups before demineralization showed higher microhardness values, i.e., nearly 300 kgf/mm^2^. Whereas after demineralization, a decrease in the microhardness values was observed. The control group shows 89.82 kgf/mm^2^ of microhardness. At the same time, isolated HA showed microhardness of about 146.68 kgf/mm^2^. After remineralization, increments in the microhardness were observed. The microhardness of the control and isolated HA was 138.6 ± 47 kgf/mm^2^ and 203.8 ± 7 kgf/mm^2,^ respectively. Several research reports are available where microhardness studies were used to examine lesion remineralization. Huang et al. have reported the In vitro lesion remineralization capability of the nano-HA and micro-HA. A microhardness study reveals that nano-HA promotes remineralization more effectively than micro-HA [54]. In another study, Juntavee et al. investigated the remineralization capability of the nano-HA gel on artificial caries lesions. The enamels’ in vitro remineralization capacity was tested by soaking them in deionized water and incubating them at 37 °C. The gel shows higher Vickers microhardness values due to the mineral deposition on the enamel surface [55].

### 3.11. The Remineralizing Potential of HA Was Assessed Using SEM-EDX Analysis

**Figure 11:**
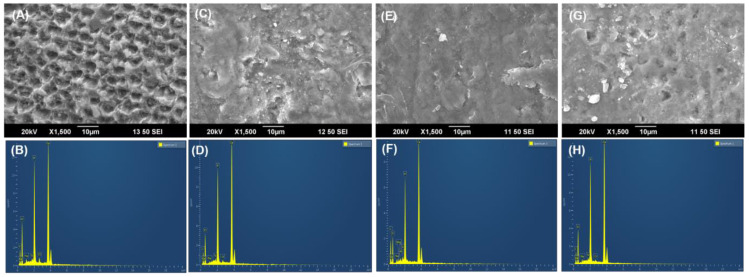
SEM (**A**,**C**,**E**,**G**) and EDX (**B**,**D**,**F**,**H**) analysis of the enamel images are the SEM images of the demineralized enamel specimen, GC tooth mousse applied enamel, alkali-treated nano HA applied enamel, and thermally calcined nano HA applied enamel, respectively.

Applied HA materials will be adhered to on the demineralized surface by nanocrystals that aggregate and develop into micro clusters, forming a homogeneous apatite layer. The newly formed apatite layer will be formed on the surface, covering the prismatic and interprismatic enamel structures. Herein, we used SEM/EDX analysis to study the remineralization potential of the studied enamels. Surface morphological observations in the SEM analysis revealed that a white apatite layer has formed on the surface of the GC tooth mousse-applied enamel (Figure 11C), alkali-treated HA-applied enamel (Figure 11E), and thermally calcined HA-applied enamel (Figure 11G). Figure 11A shows the demineralized enamel specimen. The data demonstrate that the developed thermally calcined HA and alkali-treated HA have the potential to induce mineralization, similar to well-known GC tooth mousse. EDX analysis was used to calculate the Ca to P ratio of the studied enamels. GC tooth mousse-applied enamel (Figure 11D) has a 1.80 Ca/P ratio, whereas alkali-treated HA-applied enamel (Figure 11F) and thermally calcined HA-applied enamel (Figure 11G) have a 1.99 and 1.63 Ca/P ratio, respectively. These data show that the developed thermally calcined HA and alkali-treated HA can deposit mineral content and play a vital role in dentistry [56,57]. Several studies reported that the effect of HA potentially remineralizes the caries lesion [58,59,60].

## 4. Conclusions

HA was isolated using thermal calcination and alkaline hydrolysis method from a fishbone. The physicochemical properties of the obtained HA were carried out using FT-IR, XRD, TGA, HR-TEM, and SAED analysis. FT-IR and TGA analysis demonstrated that the developed HA comprises carbonated HA, and XRD results suggest that the developed HA was coherent with JCPDS data. Alkaline hydrolysis-isolated HA has a particle size of nano-structured. Furthermore, isolated HA via the alkaline hydrolysis method was biocompatible and enhanced cell proliferation. Further, using DIAGNOdent, surface microhardness, and SEM-EDX, the remineralization potential of isolated HA on artificial caries lesions was studied. The in vitro study results show that thermally calcined HA and alkali-treated nano HA can deposit mineral content on the artificially induced early enamel lesion. In conclusion, nano HA powder extracted from fish bones benefits dentistry applications.

## Data Availability

Not applicable.
